# Radar Micro‐Doppler Signature Generation Based on Time‐Domain Digital Coding Metasurface

**DOI:** 10.1002/advs.202306850

**Published:** 2024-03-13

**Authors:** Si Ran Wang, Jun Yan Dai, Jun Chen Ke, Zhan Ye Chen, Qun Yan Zhou, Zhen Jie Qi, Ying Juan Lu, Yan Huang, Meng Ke Sun, Qiang Cheng, Tie Jun Cui

**Affiliations:** ^1^ Institute of Electromagnetic Space Southeast University Nanjing 210096 China; ^2^ State Key Laboratory of Millimeter Waves Southeast University Nanjing 210096 China; ^3^ Frontiers Science Center for Mobile Information Communication and Security Southeast University Nanjing 210096 China; ^4^ Pazhou Laboratory Huangpu Guangzhou 510555 China

**Keywords:** artificial intelligence (AI), micro‐Doppler effect, radar, time‐domain digital coding metasurface (TDCM)

## Abstract

Micro‐Doppler effect is a vital feature of a target that reflects its oscillatory motions apart from bulk motion and provides an important evidence for target recognition with radars. However, establishing the micro‐Doppler database poses a great challenge, since plenty of experiments are required to get the micro‐Doppler signatures of different targets for the purpose of analyses and interpretations with radars, which are dramatically limited by high cost and time‐consuming. Aiming to overcome these limits, a low‐cost and powerful simulation platform of the micro‐Doppler effects is proposed based on time‐domain digital coding metasurface (TDCM). Owing to the outstanding capabilities of TDCM in generating and manipulating nonlinear harmonics during wave‐matter interactions, it enables to supply rich and high‐precision electromagnetic signals with multiple micro‐Doppler frequencies to describe the micro‐motions of different objects, which are especially favored for the training of artificial intelligence algorithms in automatic target recognition and benefit a host of applications like imaging and biosensing.

## Graphical abstract

A low‐cost and high‐flexible radar micro‐Doppler signature generation platform is proposed based on metasurface. The presented metasurface contains time‐varying modulation periods, thus capable of supplying the electromagnetic signals with designable micro‐Doppler frequencies to describe micro‐motions of different objects. The proposed method is especially favored for the training of AI algorithms and benefits a host of applications like imaging and biosensing.

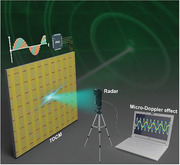

## 1 Introduction

Doppler effect describes a phenomenon, in which the carrier frequency of echoes produces a frequency shift relative to that of the incident signals when there is a relative radial motion between the source and radar target.^[^ ^1^ ^]^ Besides the bulk motion of the target, some local parts of the target also exhibit oscillatory motions, named micro‐motions, resulting in additional Doppler shifts known as micro‐Doppler effects.^[^ ^2^ ^]^ Distinguished from the Doppler shift that reflects the target velocity, the micro‐Doppler effects usually contain abundant inherent target characteristics, which are especially valued for target detection and recognition and hereby attracted extensive attention from scientists in the fields of radar, remote sensing, and medical imaging.^[^ ^3^, ^4^, ^5^ ^]^ In particular, the micro‐Doppler effects can be employed to monitor and detect human activities in both medical and secure areas. To capture high‐quality micro‐Doppler signatures, the radars usually operate in the microwave or millimeter‐wave band owing to the advantage of short wavelengths. Some advanced artificial intelligence (AI) based signal processing algorithms are also introduced to realize efficient and comprehensive information excavation from raw micro‐Doppler data.^[^ ^6^, ^7^, ^8^, ^9^ ^]^ However, to determine the relationship between the micro‐Doppler characteristics and the corresponding micro‐motions of various targets, a large number of sample data is required as the basis for offline training. By contrast, collecting sufficient micro‐Doppler signature samples is extremely time‐consuming and expensive, since massive experiments are needed with a variety of radar platforms and targets involved.

With the rapid advance of digital coding metasurfaces,^[^ ^10^ ^]^ it became possible to realize direct information modulations onto the radio‐frequency (RF) signals. As revealed by recent studies,^[^ ^11^, ^12^, ^13^, ^14^, ^15^, ^16^, ^17^, ^18^, ^19^ ^]^ when the digital coding metasurfaces are further modulated periodically in time, a set of nonlinear harmonics can be generated in the reflected spectrum. Owing to the ability of spectrum manipulations, time‐domain digital coding metasurfaces (TDCMs) have opened up a number of remarkable applications such as nonreciprocal effect,^[^ ^11^, ^12^, ^13^, ^16^ ^]^ harmonic controls,^[^ ^14^, ^15^ ^]^ efficient frequency conversion,^[^ ^17^ ^]^ and nonlinear information modulations.^[^ ^18^, ^19^ ^]^ From the radar perspective, the emergence of new frequency components in echoes may represent moving targets with radial velocities. On this basis, the feasibility of generating a controllable Doppler effect was demonstrated with a reconfigurable stationary metasurface.^[^ ^20^, ^21^ ^]^ However, the simulation of micro‐Doppler frequencies remains a great challenge because the non‐linear frequency components generated by the current TDCMs are not time‐varying.

Here, we successfully realize micro‐Doppler signal simulations with the aid of a new kind of TDCM. For the new TDCM, the frequency modulated to it is no longer time‐invariant during the matter‐wave‐information interactions, thus giving rise to the required micro‐Doppler shifts that can describe the subtle changes of the object movements. The roles of the presented metasurface in the micro‐Doppler signature generation can be divided into two aspects: 1) We can get the measured micro‐Doppler data from the experiments, and then use a low‐cost metasurface with simple architecture to generate the micro‐Doppler signals without a complicated radar signal simulator; 2) the metasurface can create the micro‐Doppler signals of new objects or even fake objects by applying various space‐time coding strategies. This is a great challenge for traditional methods and has not been achieved in the past years as far as we know. Therefore, this work takes a significant step forward in the simulation of real 3D objects, thus suggesting a new avenue for theoretical and experimental studies in the micro‐Doppler signal generation.

## 2 Theory

### 2.1 Principles of the Micro‐Doppler Effect

Assuming a frequency‐modulated continuous‐wave (FMCW) radar that is widely used to extract the micro‐Doppler features of targets,^[^ ^22^ ^]^ the corresponding transmitting chirp signal from the FMCW radar can be expressed as:

(1) $$s_{tx}\;\left(t\right)=rect\left(\frac{t}{T_{p}}\right)e^{j2\pi \left(f_{c}t+\frac{1}{2}\gamma t^{2}\right)}$$

where $rect\;\left(x\right)=\{\begin{array}{cc} 1, & |x|<0.5\\ 0, & others \end{array}$, *T_p_* denotes the duration time of a chirp signal, *B* is the bandwidth of the transmitted chirp signal, $\gamma =\frac{B}{T_{p}}$ represents the chirp rate of the transmitted chirp signal, and *f_c_* is the carrier frequency.

**Figure 1:**
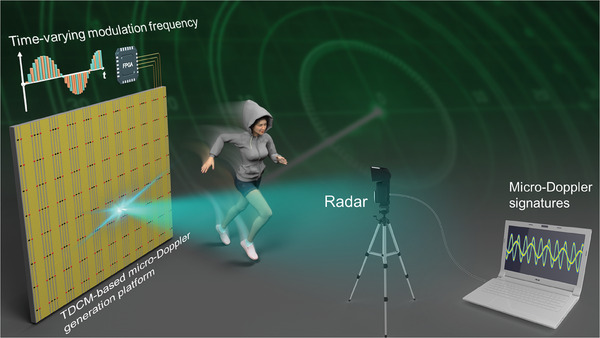
Schematic of the micro‐Doppler signature simulation with TDCM.

For a better illustration of the origin of the micro‐Doppler effect, we suppose that a person is running toward the radar with a constant pace ν at the distance of *r*_0_, as depicted in **Figure** **1**. For simplicity, the noise and clutter signals are omitted, and the amplitude of the person's echo does not change within one frame of signal. Hence, the echo signal from the moving person can be expressed as

(2) $$s_{rx}\;\left(t\right)=A\cdot rect\left(\frac{t-\frac{2r\left(\eta \right)}{c}}{T_{p}}\right)e^{j2\pi \left\{f_{c}\left[t-\frac{2r\left(\eta \right)}{c}\right]+\frac{1}{2}\gamma {\left[t-\frac{2r\left(\eta \right)}{c}\right]}^{2}\right\}}$$

in which *A* is the amplitude of the echo wave, η  =  (*l* − 1) · *T_c_* is the slow time, *l* denotes the *l^th^* chirp signal in one frame, *T_c_* is the interval between two chirps in one frame, and *r*(η) represents the instantaneous distance between the radar and the target, which can be further written as

(3) $$r\;\left(\eta \right)=r_{0}-\nu \eta +r_{mD}\left(\eta \right)$$

Here *r_mD_* (η) stands for the relative distance change caused by body micro‐motions during running, which is actually a periodic function of time.

After dechirp processing, the baseband echo signal can be approximated as^[^ ^22^ ^]^

(4) $$s_{rx-base}\;\left(f,\eta \right)=AT_{p}\cdot \mathrm{sinc}\left[T_{p}\cdot \left(f-\frac{2\gamma r\left(\eta \right)}{c}\right)\right]e^{-j\frac{4\pi f_{c}}{c}r\left(\eta \right)}$$

where *f* denotes the range frequency, and $\mathrm{sinc}\;\left(x\right)=\frac{\sin(\pi x)}{\pi x}$. We suppose that the residual video phase has been neglected for simplicity, and − (4π*f_c_*)*r*(η)/*c* reflects the additional phase caused by the Doppler effect. Furthermore, the instantaneous Doppler frequency can be given by

(5) $$f_{d}\;\left(\eta \right)=\frac{2\nu f_{c}}{c}-f_{mD}\left(\eta \right)$$

in which (2ν*f_c_*)/*c* and *f_mD_* (η) represent the Doppler and micro‐Doppler frequency shifts, respectively. As *f_mD_* (η) is a periodic signal in the time domain, it can be regarded in terms of a Fourier Series

(6) $$f_{mD}\;\left(\eta \right)=\sum_{m=1}^{\infty }b_{m}\sin\left(m\cdot 2\pi f_{s}t\right)$$

in which *b_m_* represents the amplitude of the *m^th^*‐order harmonic, and *f_s_* is the basic frequency, which always reflects the inherent property of the target.

**Figure 2:**
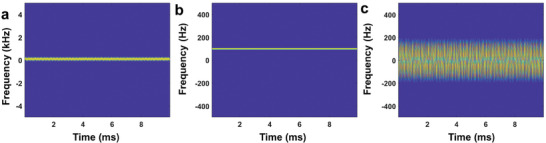
a) Time‐frequency spectrum records both Doppler and micro‐Doppler signatures of a running person. b) Time‐frequency spectrum of the corresponding Doppler signatures. The Doppler frequency is 100 Hz. c) Time‐frequency spectrum of the corresponding micro‐Doppler signatures.

In actual applications, to investigate the micro‐motions of a target, the raw data of radar echo signals are processed with the range migration and Doppler migration compensation in the time‐frequency analysis.^[^ ^1^ ^]^ **Figure** **2**a shows the time‐frequency spectrum of the running person with the Doppler and micro‐Doppler signatures. The central frequency of the radar is assumed to be 10 GHz, and the running velocity is 1.5 m s^−1^. Then the corresponding Doppler frequency can be calculated as 2*v*/λ =  100 Hz. To better observe the micro‐Doppler features of the target, the Doppler and micro‐Doppler frequency components are separately extracted, as shown in Figure 2b,c, respectively. We can find more detailed information in the micro‐Doppler figure than in the Doppler one, which is especially valued for target recognition. However, from ref. [23], it can be found that although there are large differences among micro‐Doppler spectra sometimes, it is still a great challenge to recognize its corresponding micro‐motions from a specific micro‐Doppler spectrum without the aid of expert knowledge. To overcome this difficulty, we need to set up a database that connects targets with various micro‐motion characteristics and the corresponding micro‐Doppler features. However, establishing the database will bring up new problems like large amount of time and high cost in numerous experiments. Thus, it is crucial to develop a versatile simulator that can freely mimic the micro‐Doppler features for database training.

### 2.2 Simulation of Micro‐Doppler Effects with TDCM

For a TDCM with the reflection phase altered flexibly in time domain, when it is illuminated by a monochromatic electromagnetic wave at the frequency of *f_c_*, the reflected electric field can be expressed as

(7) $$E=\left|\Gamma \left(t\right)\right|\;e^{j\left[2\pi f_{c}t+\varphi \left(t\right)\right]}$$

in which |Γ(*t*)| is the reflectivity amplitude, and φ(*t*) is the reflection phase continuous and differentiable over the time. For a highly reflective metasurface we usually have |Γ(*t*)| =  1. Based on Equation (5), the instantaneous reflected frequency of the metasurface can be expressed as the derivative of the reflection phase, that is

(8) $$f=\frac{1}{2\pi }\;\frac{d\varphi \left(t\right)}{dt}+f_{c}$$

If a constant modulation period *T*_0_ is used for TDCM, Equation (8) can be further modified as

(9) $$f=\frac{1}{2\pi }\;\frac{\varphi \left(T_{0}\right)-\varphi \left(0\right)}{T_{0}}+f_{c}$$

To ensure the phase continuity during the modulation, φ(*T*_0_) − φ (0) =  2*m*π, wherein *m* is an integer.^[^ ^17^, ^19^ ^]^ Hence the instantaneous reflected frequency is *m*/*T*_0_  + *f_c_* from Equation (8). In most of the reported TDCM designs, the modulation period *T*_0_ does not change and is fixed, resulting in the fixed frequency offsets for all harmonics. For instance, when *m*  =  1 and *T*_0_ =  1 us, the corresponding frequency offset between the reflected and incident waves will be 1 MHz. Note that TDCM can only simulate the Doppler effect, but fails to provide high‐frequency components representing the micro‐Doppler effect as revealed in Figure 2. This can be understood since the instantaneous reflected frequency *m*/*T*_0_  + *f_c_* remains constant when m and *T*_0_ are fixed. In this case, only a Doppler frequency component *m*/*T*_0_ can be achieved.

In order to obtain the time‐varying frequency offset as the summation of the Doppler and micro‐Doppler frequency components, we propose to employ a time‐varying modulation period *T*(*t*), where the echo frequency becomes

(10) $$f\left(t\right)=\frac{1}{2\pi }\;\frac{\varphi \left(T\left(t\right)\right)-\varphi \left(0\right)}{T\left(t\right)}+f_{c}$$

**Figure 3:**
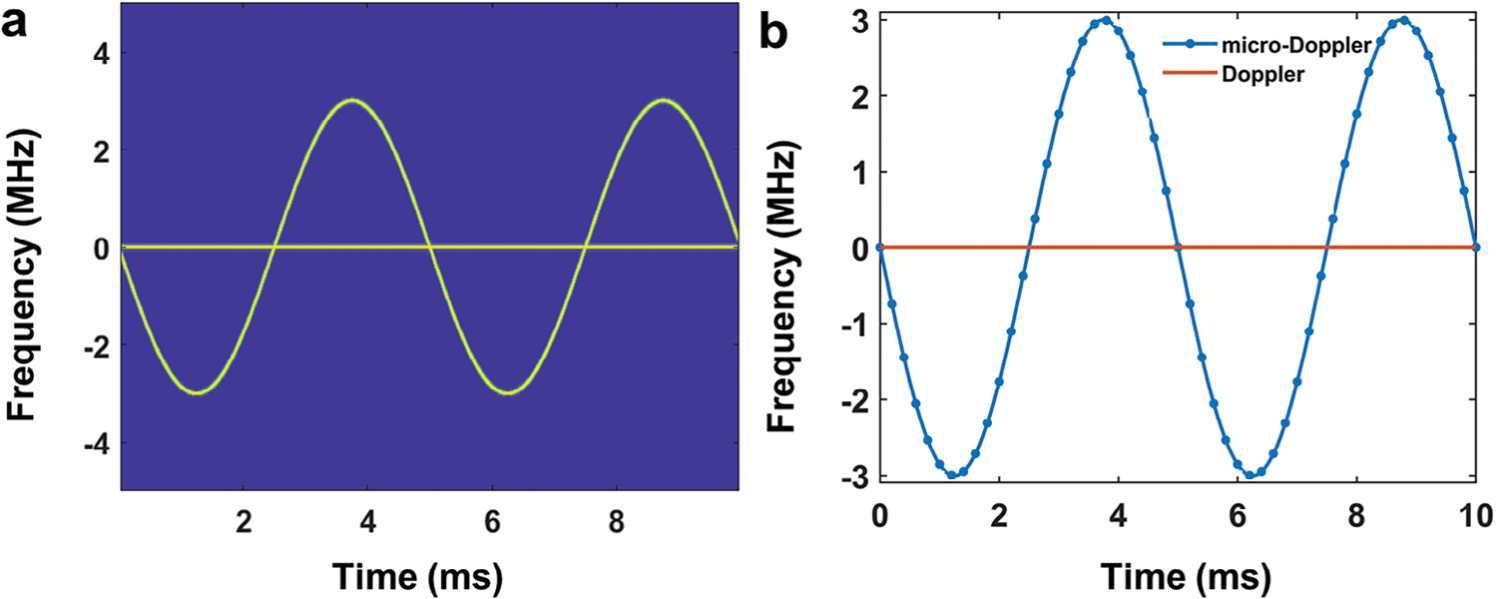
a) Time‐frequency spectra of the Doppler and micro‐Doppler signals generated by TDCM. b) The Doppler and the micro‐Doppler components extracted from (a). The micro‐Doppler and Doppler frequencies are 3e6 × sin(400πt) Hz and 100 Hz, respectively.

**Figure 4:**
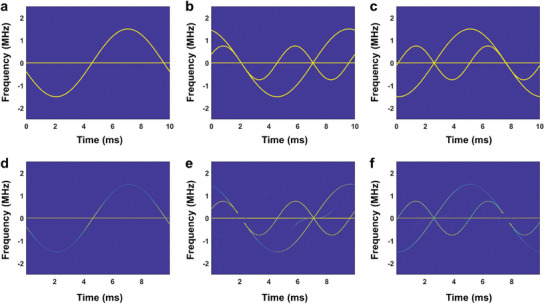
Comparison of simulated and measured sinusoidal micro‐Doppler and constant Doppler signals generated by the TDCM sample, in which the signal parameters described in Case I to Case III are presented in Table 1, respectively. a–c) The simulation results. d–f) The measured results.

It offers the possibility to give rich frequency components for the Doppler and micro‐Doppler effect simulations. As an example, we assume that the instantaneous reflection frequency is a sinusoidal function of time

(11) $$f\;\left(t\right)=A\sin\left(\omega t+\varphi \right)+f_{0}$$

where *A*, ω, and φ represent the amplitude, angular frequency, and the initial phase, and *f*_0_ is the Doppler frequency. The carrier frequency is omitted. Here, we choose *A*  =  3 *MHz*, ω  =  2π × 200 *Hz*, φ  =  0 and *f*_0_ =  100 *Hz*. For better illustration, the Doppler and micro‐Doppler signals are separated from **Figure** **3**a, which are presented in Figure 3b,c, respectively. In addition, as multiple micro‐motions may appear simultaneously in the same target, the instantaneous reflection frequency can be expressed as

(12) $$f\;\left(t\right)=\sum_{i=1}^{n}A_{i}\sin\left(\omega_{i}t+\varphi_{i}\right)+f_{0},$$

where the amplitude and phase are differed due to the relative positions of the parts with micro‐motions. For simplicity, we consider a moving target containing two kinds of micro‐motions with *n*  =  2. The Doppler frequency is set to be 100 *Hz*, corresponding to a moving target with the velocity *v* = 1.5 m s^−1^. Owing to the parameter variance in Equation (12), we consider three cases that stand for dual micro‐motions with different postures (see **Table** **1**), and the corresponding calculated time‐frequency curves can be found in **Figure** **4**a–c.

**Table 1:** Parameters of the generated micro‐Doppler and Doppler signals in Equation (12).

	*f*_0_[Hz]	*A*_1_[MHz]	*A*_2_[MHz]	ω_1_[rad s^−1^]	ω_2_[rad s^−1^]	|φ_1_ − φ_2_|[rad]
Case I	100	1.5	1.5	200π	200π	0
Case II	100	1.5	0.75	200π	400π	π/2
Case III	100	0.75	1.5	400π	200π	π/2

## 3 Experimental Section

**Figure 5:**
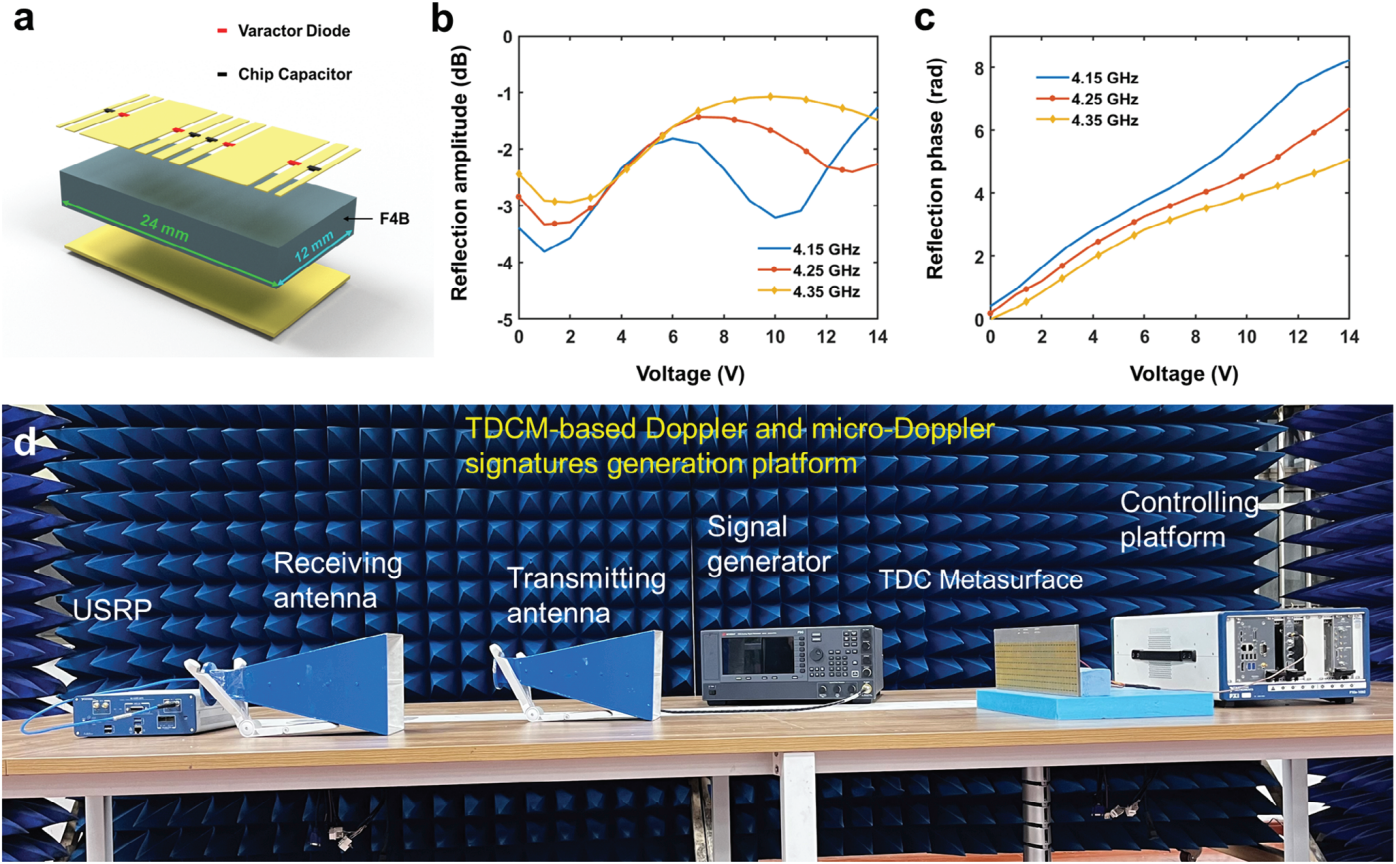
a) Exploded view of the meta‐atom. b,c) Reflection phase and amplitude spectra of the meta‐atom with the change of biasing voltage at 4.15, 4.25, and 4.35 GHz. d) Experimental configuration of the platform.

Since the micro‐Doppler effect is critical to help researchers recognize the target micro‐motions, while the Doppler effect is only related to the target velocity, in the experiment we mainly focus on the generation of micro‐Doppler frequencies. To evaluate the feasibility of the theoretical analysis, a TDCM sample composed of 16 × 8 meta‐atoms is employed to change the reflection phase continuously over time. The meta‐atom is a three‐layered structure (**Figure** **5**a), with the top and bottom copper layers sandwiched by the middle F4B (ε_*r*_ =  3.0 tan σ =  0.0015) substrate. In the top pattern, four chip capacitors (in black) and four varactor diodes (in red) are used to bridge adjacent metallic strips. The thickness, length, and width of the meta‐atom are 5 mm, 24 mm, and 12 mm, respectively.^[^ ^24^ ^]^ The commercial software package, CST Microwave Studio 2016, is used to monitor the reflectivity variation with the change of diode biasing voltage at 4.15, 4.25, and 4.25 GHz, respectively. In the simulations, the frequency domain solver is adopted. The number of mesh cells is 56 962, and the boundary condition is unit cell. The details of the simulations have been added to the revised manuscripts. From Figure 5b, we see the simulated amplitude fluctuation <4 dB, along with the full phase range achieved with the growth of the biasing voltage. The latter is especially important to ensure phase continuity during the modulation as stated above.

Two experiments are carried out to demonstrate the capability of micro‐Doppler frequency generation and simulation. Figure 5d shows the details of the experimental configuration, where the fabricated TDCM is temporally modulated using a controlling platform (provided by NI Corp). Specifically, the platform is composed of a high‐speed I/O bus controller, an FPGA module, a digital‐analog conversion module, a DC power supply module, and a timing module. During the experiment, the metasurface is excited by a linearly polarized antenna connected to a microwave signal generator at 4.25 GHz. At the receiving end, another horn antenna is connected to a universal software radio peripheral (USRP) placed to record signals reflected from the TDCM. Here, the USRP is used to demodulate the baseband signals containing micro‐Doppler features.

First, the TDCM sample is used to generate the micro‐Doppler characteristics with the corresponding time‐frequency curves described by sinusoidal functions. TDCM is modulated with the periodic signals, in which the time‐varying phase and the period can be determined in Equation (10). The measured Doppler and micro‐Doppler signals via the proposed TDCM are presented in Figure 4d–f, where the three cases in Table 1 are considered for comparison. It is evident that the experimental results are in good accordance with the calculated ones. As shown in Figure 4e,f, some undesired frequency components exist in the measured spectra, which may be primarily attributed to the non‐ideal reflection amplitude responses for the metasurface in the presence of the resonance loss. Furthermore, the imperfect controlling waveforms of the meta‐atoms will also lead to the emergence of spurious signals.

**Figure 6:**
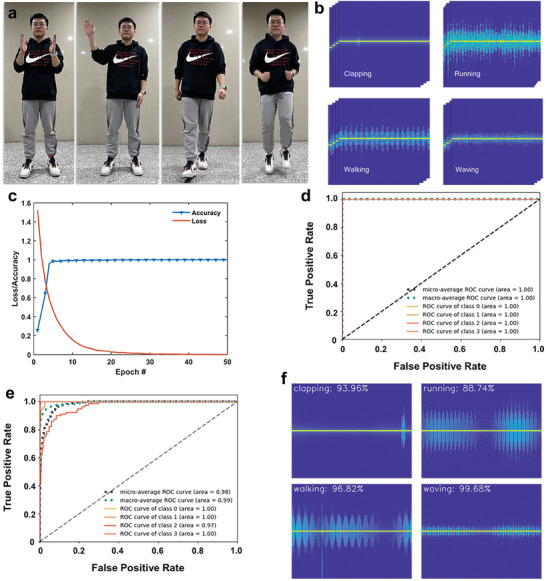
a) Photographs of four typical actions including clapping, waving, walking, and running. b) The measured time‐frequency spectra of the four actions with the FMCW radar. c) Loss and accuracy of the trained CNN. d) AUC‐ROC of the trained CNN with real data. e) AUC‐ROC of the trained CNN with constructed data simulated by the TDCM. f) Classification of the simulated time‐frequency spectra of the four actions with TDCM.

Second, the TDCM sample is employed to generate the micro‐Doppler characteristics of a human body. Unlike the micro‐Doppler characteristic curves in the previous experiments, sometimes the micro‐Doppler signals of human actions cannot be represented by an analytic way. Therefore, we employ a convolutional neural network (CNN)^[^ ^24^ ^]^ to test the feasibility of the generated micro‐Doppler signals with the aid of metasurface. To acquire enough training data, we capture the body micro‐motion signals repeatedly by an FMCW radar with the bandwidth of 400 MHz. **Figure** **6**a shows four typical actions for micro‐Doppler signal extractions, including waving, clapping, walking, and running. Micro‐Doppler time‐frequency curves were finally obtained after a series of signal processing operations such as matched filtering and time‐frequency analysis, as illustrated in Figure 6b.

Based on the complexity of micro‐Doppler characteristic images, 60% data was used for CNN training, 20% for CNN validating, and 20% for CNN testing. The ground truth labels of the four micro‐Doppler characteristic images are clapping (class 0), running (class 1), walking (class 2), and waving (class 3). The architecture of the CNN model, the activation function, the loss function, and the optimizer are ResNet‐18,^[^ ^25^ ^]^ ReLU, cross‐entropy, and Adaptive Moment Estimation (Adam), respectively. As illustrated in Figure 6c, the loss value declines until it slows down. To avoid over‐fitting, the training was stopped after around 50 epochs. Then, the test set was used for evaluating the performance of the CNN models. We adopted the macro‐average method to calculate the precision, recall, and f1‐score which are 100%, 100%, and 100%, respectively. The AUC‐ROC curves of the four images are shown in Figure 6d. The AUC is the area of the ROC curve. The evaluation results indicate that the trained CNN shows good classification ability. From the viewpoint of radar, the amplitudes and repetition periods of the micro‐Doppler characteristic curves are to some extent proportional to the vigor of the motions. The micro‐Doppler images of the clapping and waving are quite different from those of walking and running. Therefore, it is enough to adopt a generalized ResNet‐18 model for classification.

Next, we proceed to prove the micro‐Doppler characteristic simulation ability of TDCM via the trained CNN. Here, we use artificially generated micro‐Doppler data simulated by TDCM as the source signal for transmission, where the signals are superpositions of sine functions with different amplitudes, phases, and periods. Then the signals are received by USRP and processed, which are used as the input of CNN as a new test set.

In CNN, the precision, recall, and f1‐score calculated with the new test set are 90%, 87%, and 87%, respectively. The corresponding AUC‐ROC curves are illustrated in Figure 6e. Moreover, a set of micro‐Doppler characteristic images with predicted classification precisions is illustrated in Figure 6f. It can be seen that the micro‐Doppler characteristics simulated by TDCM can be classified correctly by CNN. The predicted classification precisions are not 100% owing to the deviations of frequency components between the artificially constructed and measured signals. Therefore, to ensure that the generated curves are close to the real ones, the modulation signal to the metasurface should include more high‐frequency sine components.

## 4 Discussion

We investigate the possibility of the micro‐Doppler signature generations with the TDCM. By changing the time‐varying modulation period and phase distributions, we show that TDCM can simulate the micro‐Doppler signals from multiple micro‐motions of a moving target, thereby offering a direct and efficient tool to aid target recognition of different radars. In the experiment, high recognition probability is demonstrated when the TDCM‐based micro‐Doppler signals are recorded by the receiver and processed by CNN for classification, which is in good agreement with the theoretical predictions.

## Conflict of Interest

The authors declare no conflict of interest.

## Data Availability

The data that support the findings of this study are available on request from the corresponding author. The data are not publicly available due to privacy or ethical restrictions.
